# Sports Participation and Osteoarthritis in Females: A Systematic Review †

**DOI:** 10.3390/sports12010015

**Published:** 2023-12-31

**Authors:** Magnus Brent, Mikkel Bo Brent

**Affiliations:** 1 Department of Health Science and Technology, Aalborg University, 9200 Aalborg Øst, Denmark; 2 Department of Biomedicine, Health, Aarhus University, 8000 Aarhus, Denmark

**Keywords:** osteoarthritis, elite sports, non-elite sports, Olympic sports, bone

## Abstract

Sports participation and the risk of osteoarthritis (OA) have been a concern for decades. Few research efforts have been dedicated to clarify this issue for females, although they are considered at greater risk of developing OA than males. In contrast, several reviews have established an association between sports participation and OA for males. The aim of the systematic review was to assess the association between OA and participation in popular sports for females. PubMed, Embase, and Google Scholar were searched and yielded 578 articles. Nine eligible studies were included and covered ballet (age range: 19–54 years), running or tennis (age range: 40–65 years), Olympic sports (age range: not specified), volleyball (age range: 16.0 ± 0.8 to 46.8 ± 5.1 years), and cross-country skiing (age range: 15 to ≥60 years). For females, participating in sports at an elite level was associated with a higher risk of OA and an increased need for surgical treatment. At non-elite level, it was associated with a higher risk of OA, but it did not materialize to an increased risk for surgical treatment. Few studies compared females and males, and these studies suggested that sex did not affect the risk of developing OA from participating in sports. Nevertheless, to isolate the precise effect of sports participation on the development of OA remains difficult as injuries are common among athletes and are independently associated with an increased risk of OA.

## 1. Introduction

Osteoarthritis (OA) is the most common form of arthritis and is a leading cause of pain and disability [1]. The disease represents a growing burden for healthcare systems, societal economy, and the individuals who are affected [2,3]. This is underlined by a global burden of disease study that systematically analyzed OA prevalence in 195 countries from 1990 to 2017. The study found an increasing prevalence in most countries and the increase in OA is expected to continue with longer life expectancy and ageing [4]. A recent study conducted in Denmark examined the prevalence rates of 199 chronic diseases and ranked OA as the ninth most common chronic disease [5]. The economic burden of the disease is highlighted by OA being the fifth most expensive disease treated in USA costing USD 12.8 billion with 229,000 stays in hospitals per year [6]. Moreover, a Swedish cohort study has reported that patients with physician-diagnosed OA had an approximately 50% increased risk of receiving disability pension and a two-fold increased risk of receiving sickness benefits [7].

OA is characterized as a degenerative joint disease that involves a complex interaction between multiple tissues [8]. OA is generally recognized as a disease with a slow biologic progression with distinctly different stages [9]. The pathogenesis of OA is not fully elucidated, but several underlying cellular and molecular mechanisms have been suggested to play a key role in the progression of OA. These mechanisms can promote increased degenerative changes of the articular cartilage, structural changes in the subchondral bone, osteophytes at the bone surface, bone marrow lesions (BML), cysts, sclerosis, synovitis, and meniscal tears [10].

The main symptoms of OA include pain, swelling, morning stiffness, crepitation, and reduced physical function [11]. The symptoms of pain and reduced physical function are associated with impaired functional independence in adults and elderly individuals diagnosed with OA [12]. This includes limitations in daily activities such as chair and bed transfers, dressing, and stairclimbing. In addition, females with OA have also shown higher rates of anxiety, depression, and lower quality of life when compared to controls [13].

In the last decade, important insights have been gained into the risk factors for OA development and treatment strategies to reduce pain and increase movement [14,15]. Studies have identified age [16], sex [17], ethnicity [18], genetics [19], nutrition [20], obesity [21], and occupational activity level [22] as risk factors for the development of OA. Physical exercise, as a non-pharmacological treatment, is commonly encouraged in several national guidelines as a first-line treatment for OA [23,24,25]. This is based upon the principle that exercise can improve joint flexibility and stability and reduce pain, thereby achieving functional capacity and independence [26]. Despite positive outcomes, vigorous-intensity exercise entails a risk of exercise-related injuries in joints [27]. The damaged joint can cause alterations in articular cartilage, ligaments, and menisci that may not be initially seen until months or even years later and consequently initiate OA development [28,29,30].

The relationship between sports participation and OA has demonstrated conflicting results. A recent systematic review conducted by Bestwick-Stevenson et al. (2021) investigated the association between OA and males participating in popular North American sports [31]. They found that overall sports participation was associated with an increased risk of OA, but the risk differed by individual sports. Similar results have been found in a systematic review conducted by Tran et al. (2016) [32]. They investigated the association with participation in the 32 most popular sports in England and the risk of developing OA in males. They found that OA development was associated with elite sports participation in the majority of the sport types examined, but it was unclear whether non-elite sports participation was associated with OA development. However, females are in general at greater risk of developing OA in knees [33], hips [34], and hands compared with males [35], but very little is known about how sports participation impacts the risk of developing OA in females.

Therefore, the main aim of the present systematic review was to investigate the association between OA development and participation in popular sports in Europe or North and South America among females. In addition, the impact of sex and the risk of OA and sports participation will be assessed briefly based on studies reporting data from both females and males.

## 2. Materials and Methods

### 2.1. Search Strategy

The two databases, PubMed and Embase, were searched systematically from their inception to 29 September 2022 in order to identify studies investigating the association between female sport participation and OA. To further strengthen the search strategy, backwards and forwards citation chaining was performed on all included articles to identify relevant studies not found in PubMed and Embase [36]. The search string was constructed by five individual blocks, where each block represented a specific theme. The complete search string for PubMed and Embase is available in Appendix A (Table A1). Block one consisted of keywords for biological sex (female). Block two included synonyms and spelling variations of OA. Block three was composed of popular sports in Europe and North and South America. Block four consisted of common anatomical sites for OA including both weight bearing joints (knee, hip, ankle, spine, and neck) and non-weight bearing joints (elbow, shoulder, and hand). Block five included study designs of interest to investigate the association between sport and OA development. Blocks two to four were inspired by two similar systematic reviews by Bestwick-Stevenson et al. (2021) and Tran et al. (2016) [31,32]. Finally, Google Scholar was free text searched to identify additional records not indexed in the two primary databases. No formal search string was developed for Google Scholar, but the same keywords presented in Block 1 and Block 2 (Table A1) were used in various combinations. The overall search strategy and methodology were developed based on experiences from previous reviews [37,38,39,40].

### 2.2. Definitions and Eligibility Criteria

To distinguish between sport, physical activity, and exercise, the following definitions were used: Sport was defined as a subset of exercise that can be undertaken individually or as a part of a team [41]. Participants adhere to a common set of rules or expectations, and a defined goal exists. Physical activity was defined as any bodily moment produced by skeletal muscles that results in energy expenditure [42]. Exercise was defined as planned, structured, and repetitive bodily movement, the objective of which is to improve or maintain physical fitness [42]. As there is no clear definition of elite level across all sports included in this review, the performance level was categorized into non-elite or elite. The term elite was used if sports participation was performed at either professional, national, or international level. Grey literature was defined as literature or data produced outside of traditional academic publishing and distribution channels i.e., working papers, newsletters, government documents, reports, etc. [43]. In order to standardize the abstract and full-text screening process, a prioritized list of exclusion criteria was used in the screening process of titles, abstracts, and full-text articles. No study design restrictions were applied as eligibility criteria for inclusion. Screening and data extraction were performed by MB.

1. Not available in English.
2. No available data on OA.
3. Not investigating sports participation.
4. Not reporting specific outcomes for females.
5. Not review articles or grey literature.

Data were retrieved using an extraction template with the following headlines:

Name of the first author; publication year; study design; authors conclusion; anatomic joint assessed; diagnosis of OA; incidence; risk or prevalence of OA; performance level (elite or non-elite); type of sport; demographics (age, body mass index, duration of competitive career, age when starting to compete, age when ceasing to compete); surgical treatment; and sample size.

The review was not registered in an online protocol repository, however, the definition of OA, search strategies, and a detailed data extraction protocol were created prior to performing the search.

The systematic review was conducted and reported in accordance with the Preferred Reporting Items for Systematic Reviews and Meta-Analyses (PRISMA) guidelines [44]. The PRISMA Checklist is available online as supplementary material.

### 2.3. Risk of Bias Assessment

Choosing a suitable methodological quality (risk of bias) assessment tool is an important task allowing transparency of included studies by a critical appraisal in systematic reviews [45]. Since the present systematic review included different types of observational study designs (cohort, case-control, and cross-sectional), two different assessment tools were used to assess methodological quality of the included studies. To assess the quality of observational cohort and cross-sectional studies, the National Institutes of Health (NIH) quality assessment tool was used (https://www.nhlbi.nih.gov/health-topics/study-quality-assessment-tools, accessed 1 December 2022) [45] (Table S1—Supplementary Materials).

The assessment tool includes 14 different items with rating scores of “yes”, “no”, or “other” (cannot determine (CD), not applicable (NA), or not reported (NR)). An overall score between 0 and 5 points was considered a high risk of bias, a score between 6 to 9 points indicated a fair risk of bias with some flaws, and a score > 10 points signified little or no risk of bias. To assess the quality of observational case-control studies, the Scottish Intercollegiate Guidelines Network (SIGN) methodological assessment tool was used (https://www.sign.ac.uk/what-we-do/methodology/checklists/, accessed 1 December 2022) [45] (Table S2—Supplementary materials). This tool includes 15 items to evaluate internal validity with four subheadings (selection of subjects, assessment, confounding, and statistical analysis), and lastly an overall assessment of the study with a rating score including “yes”, “no”, or “can’t say”. An overall score between 0 and 5 points was considered a high risk of bias, a score between 6 and 10 points indicated an acceptable study quality and an associated risk of bias, and a score >10 points was considered as little or no risk of bias. It should be noted that two different bias assessment tools were used and a holistic judgement of risk of bias should be viewed with caution. The risk of bias assessment was performed by MB.

### 2.4. Study Selection and Data Extraction

**Figure 1:**
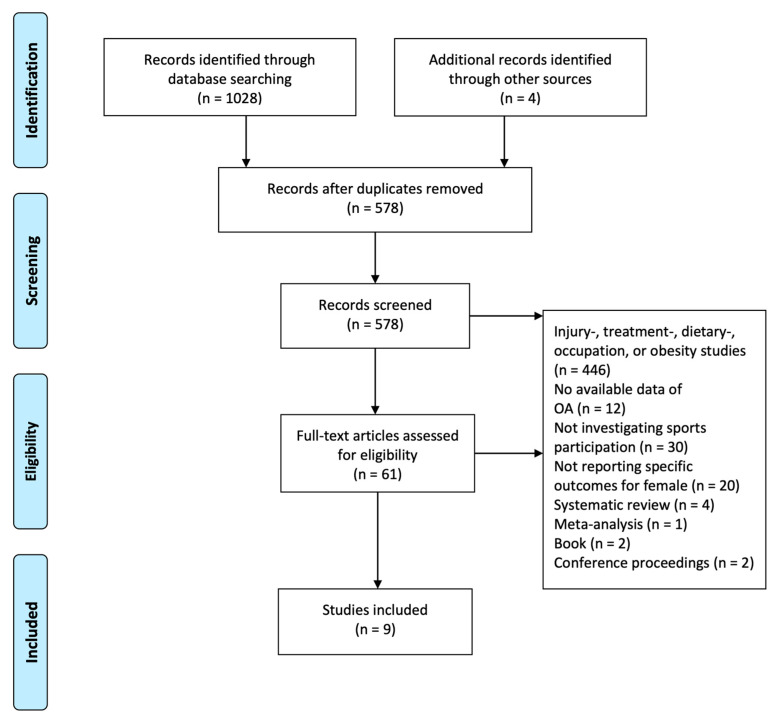
PRISMA flow chart. Injury studies were used as a broader term, including tear of the anterior cruciate ligament (ACL), meniscal tears, intra-articular fractures, and sacral fractures. Treatment studies were used as a broader term, including intra-articular injections, pharmacological management, osteotomy, arthroscopy, and arthrodesis.

A total of 1028 study records were identified through PubMed and Embase, while two additional study records were identified through backwards reference chaining. Two other studies were identified through free text search of Google Scholar (Figure 1).

454 records were discarded as duplicates. Of the remaining 578 unique studies, 517 were excluded through title and abstract screening. Then, 61 studies were full-text assessed and, consequently, a total of nine eligible studies were included for analysis (Figure 1). The nine included studies were conducted in the United Kingdom (UK), Germany, Sweden, Netherlands, United States (US), and Brazil. There was no outcome parameter common to all nine studies since several different diagnostic methods were used, including questionnaires [46,47], measured markers of OA using magnetic resonance imaging (MRI) [48,49,50] and radiography [51,52,53], or surgery and arthroplasty data collected through national health registers [54]. Demographic and arthroplasty data from the included studies are presented in Table 1.

### 2.5. Data Synthesis

All included studies were encompassed in the final data synthesis. Data were extracted and summarized in tables. In case of missing data or pooled data for both females and males, authors were contacted by mail to request the full data sets.

## 3. Results

The anatomical sites assessed were as follows: knee (89%) [46,47,48,49,50,51,53,54], hip (78%) [46,47,48,50,51,52,54], ankle (44%) [47,48,50,52], spine (33%) [47,50,53], foot (33%) [47,50,52], hand (22%) [47,53], shoulder (11%) [48], elbow (11%) [48], and wrist (11%) (Table 2) [48]. Seven of the included studies recruited elite sports participants, with a total of 808 females [46,47,48,49,50,51,52], and two studies recruited non-elite sports participants, with a total of 5427 females (Table 1) [53,54]. The study designs were: four cross-sectional studies (44%) [46,47,48,49], three case-control studies (33%) [51,52,53], and two cohort studies (22%) [50,54].

### 3.1. Ballet

Two studies investigating the association between OA and ballet dancing were included. The study conducted by Angioi et al. (2014) recruited eleven female and four male active dancers with an age range of 19–36 years [50]. The female dancers had been dancing at a professional level <5 years with a mean dancing time of 45 h per week. Participants were included if they experienced chronic pain or discomfort in the foot, ankle, hip, spine, or knee, and dancers that were already diagnosed with OA. MRI images were obtained from the anatomical sites of chronic pain or discomfort to evaluate joint space narrowing, bone marrow changes, osteophytes, cysts, and subchondral sclerosis. The Kellgren–Lawrence (KL) scale was used to evaluate the presence of knee OA (Table S3—Supplementary Materials). That study identified seven female dancers with early signs of OA development. Two dancers had KL grade two signs of OA in the right knee, and three dancers exhibited grade one signs of OA in the left knee. However, no dancer demonstrated bilateral signs of knee OA. Finally, one dancer showed early signs of OA in the lumbar spine at facet joints of L5–S1 and one dancer showed a deep recess of frayed labrum and joint space narrowing in the right hip [50].

The study conducted by Niek Van Dijk et al. (1995) recruited 19 former professional dancers with long careers, averaging 37 years (range: 13–54 years), and a mean age of 59 years (range: 50–66 years) [52]. The mean dancing time was estimated to 45 h (range, 10 to 70 h) per week when the dancers were still competing. Two of the dancers were already diagnosed with hip OA and have had surgery. The dancers were compared with non-ballet dancers who were matched to body weight, age, and height. Radiographs of the ankle, subtalar, hip, and first metatarsophalangeal joints were assessed. Measures of joint space and evaluation of subchondral sclerosis, bone destruction, osteophytes, and cysts were evaluated. The data were finally used to classify OA and grade the severity from I–III using the modified scale of Hermodsson [55].

Seven dancers had grade I OA in the right subtalar joint and it was significantly more common compared to the control group. Nine dancers had grade I and two dancers had grade II OA in the ankle. The dancer’s first metatarsophalangeal joints showed several cases of OA, including fourteen cases of grade I, seven cases of grade II, and eight cases had grade III joint changes. OA in these joints were significantly increased in dancers compared to controls. In ballet dancers, the right ankle and right first metatarsophalangeal joint spaces were smaller in width compared with controls. For hips, thirteen cases of OA were found in ballet dancers and only eight cases in the control group. However, this difference was not found to be statistically significant. Finally, 17 of the dancers had no joint complaints or symptoms of OA [52].

In summary, these findings suggest that professional ballet dancers frequently show signs of OA and are more prone to developing OA compared to matched controls.

### 3.2. Volleyball

The study by Boeth et al. (2017) examined the association between competitive high-level volleyball players and knee abnormalities in a longitudinal cross-sectional study over a two-year period [49]. A total of 36 adolescents (*n* = 10 females) and adults (*n* = 9 females) were recruited. Prior injury treatments in the female adults included one knee arthroscopy, one patella surgery, one medial meniscectomy, and one meniscus surgery. Prior injury treatment reported in one of the female adolescents included in the study was a single anterior cruciate ligament (ACL) reconstruction. The athletes trained 24 h per week using an Olympic training program. Data were obtained using MRI scanning at different regions of the knees including the patellofemoral joint (PFJ), medial femorotibial joint (MFTJ), and lateral femorotibial joint (LFT). The structural pathology of the knees was analyzed using the whole-organ magnetic resonance imaging score (WORMS) to assess prevalence and severity of subarticular bone marrow lesions (BML), osteophytes, cartilage status, ligament and meniscus integrity, and subarticular cysts. It should be noted that the study did use pooled data (females and males combined) for some of the statistical analyses, hence it was not possible to extract results exclusively from females only for all parameters investigated.

Several early signs of OA were reported at baseline; 21% had cartilage abnormalities, 26% had BML, 63% had osteophytes, and 16% had subarticular cysts. At follow-up, the mean severity for cartilage abnormalities ranged between full-thickness focal defects smaller than 1 cm at the largest width and multiple areas of partial thickness smaller than 1 cm at the largest width. The size of osteophytes was small and the severity of subarticular BML and cysts was smaller than 25% of the region. From baseline to follow-up, new signs of early OA were discovered in knee joints including two BMLs (37%) and one incident of osteophytes (68%) among all included participants.

Interestingly, no significant differences were found between females and males. No significant differences were found from baseline to follow-up for prevalence or severity of knee OA in both sexes. The severity of knee abnormalities was not significantly different between females and males in any of the specific regions assessed. Moreover, a greater prevalence of meniscal lesions was observed in adults with prior knee injury compared to adults without prior knee injury at baseline, but not at follow-up. The severity of meniscal lesions was greater in prior knee injury adults at baseline and also at follow-up compared to adults without injuries, and finally compared to all participants in each group without knee injuries. Osteophytes were observed to be more severe at follow-up in adults with injuries compared to adults without injuries, and all participants in each subgroup. No significant difference was found at baseline between prior injured adults compared to all participants without prior injuries in each subgroup. Lastly, no significant difference was found in prevalence of other knee abnormalities in prior injured adults compared to all participants without prior injuries [49].

In summary, these findings suggest that early OA were present in up to 68% of female elite volleyball players and the prevalence and severity increased in adults compared to adolescents. However, no overall difference was found between females and males.

### 3.3. Olympic Sports

Three studies investigated the association between Olympic athletes and OA. The study conducted by Merritt et al. (2021) examined the frequency and severity of OA in active athletes during the Olympic Games in Rio 2016 [48]. The study included both males and females with a total of 320 participants (*n* = 160, females). The athletes underwent MRI examinations on the following joints: ankle, knee, hip, shoulder, elbow, and wrist. The severity of OA was defined by three different grades (mild, moderate, and severe) by the presence of increasing severity of cartilage damage and the presence of osteophytes. It should be noted that the study used pooled data (females and males combined) for some of the statistical analyses, hence it was not possible to extract results exclusively from females only for all parameters investigated.

OA was almost equally distributed in females and males (16% and 18%, respectively) and was not significantly different from each other. The assessed severity of OA in females was most frequently graded as severe (7.5%) compared to both moderate (4.4%) and mild (3.8%) OA for all joints combined. A similar pattern was observed in males. The wrist was the joint most affected by OA (29%), followed by knee (23%), elbow (14%), shoulder (13%), ankle (11%), and hip (2.5%) for females and males combined. Increasing age was statically associated with OA (data from females and males combined) [48].

Two studies conducted by Cooper et al. (2018 and 2021) investigated the prevalence of OA among retired Olympic athletes [46,47]. The first study investigated the prevalence of OA in the knee and hip, and several factors associated with OA and pain at these anatomical sites. The study included both males and females with a total of 605 participants (*n* = 244 females). A survey was used to collect data about demographics, medical history, pain, presence of physician-diagnosed OA, joint replacement, joint flexibility, knee alignment, and history of significant injuries.

The prevalence of OA diagnosed at any joint in females was 26%, and 3.8% were treated with arthroplasty of both the knee and hip. Perceived pain at any joint for most days of the last month was more frequently observed in females (69%) than in males (64%). Females also had a greater incidence of retirement in their sports due to injuries compared to males (23% and 16%, respectively). For pooled data for both females and males, factors associated with knee pain included older age, obesity, widespread pain, knee injury, and weight-bearing sports participation. Knee OA was associated with several factors such as obesity, older age, knee injury, and varus knee malalignment. Hip pain was associated with widespread pain, weight bearing sports participation, and prior injury. Hip OA was associated with older age and prior hip injury [46].

The second study investigated the prevalence of OA, pain, and injuries in several anatomic sites (cervical and lumbar spine, 1st carpometacarpal joint, hip, knee, ankle, and 1st metatarsophalangeal joint). The study included both males and females with a total of 650 participants (*n* = 274 females). A survey was used to collect data including demographics, medical history, pain, presence of physician-diagnosed OA, injuries, pain, and joint replacement surgery.

The prevalence of diagnosed OA was highest in the knee 12% (95% confidence interval (CI): 7.7–15.4), hip 7.8% (95% CI: 4.6–11.1), and lumbar spine 7.5% (95% CI: 4.3–10.6). Lower prevalence of diagnosed OA was found in the ankle 2.2% (95% CI: 0.5–4.0), the 1st metatarsophalangeal joint 1.9% (95% CI: 0.2–3.5), the 1st carpometacarpal joint 2.2% (95% CI: 0.5–4.0), and in the cervical spine 1.9% (95% CI: 0.2–3.5). A similar pattern of pain was reported for the different anatomical sites investigated. A total of 369 injuries (1.4% per participant) were reported and surgical treatment with joint replacement due to severe OA were reported (knee arthroplasty: 3.8% and hip arthroplasty: 5.3%). For pooled data for both males and females, the study found that injury was the main attributable factor associated with OA, pain, and joint replacement [47].

In summary, these findings suggest that competitive sports at an elite Olympic level are associated with developing OA. Wrist and knee were the most affected joints, and the disease seems to be influenced by several other factors such as history of injuries, age, and weight bearing sport.

### 3.4. Tennis and Running

The study conducted by Spector et al. (1996) investigated the association between long-term weight bearing sports and hip and knee OA [51]. The study recruited 81 retired elite female athletes, including 14 tennis players, 67 middle- and long-distance runners, and a control group consisting of 1003 females. Radiographs of the hip and knee (patellofemoral and tibiofemoral joints) were obtained and the presence of joint space narrowing and osteophytes was measured. Osteophytes were graded on a 0–3 scale, where 1 was equivalent to KL grade 2 (Table S3—Supplementary Materials). The severity of joint space narrowing was graded from 0–3.

In the tibiofemoral joint, the frequency of osteophytes was higher in the athletes (22%) compared to the control group (15%). Tennis and running were associated with a two-fold increased risk of developing osteophytes compared to controls (odds ratio (OR): 2.1, 95% CI: 1.2–3.7). Surprisingly, the frequency of joint space narrowing in the tibiofemoral joint was higher in the controls (37%) compared to the athletes (35%) (OR 0.9, 95% CI: 0.6–1.5). In athletes, the patellofemoral joint more frequently showed osteophytes (42%) compared to controls (28%), and the risk was almost three-fold higher for running and tennis (OR 2.7, 95% CI: 1.5–5.1). Development of joint space narrowing was highest in athletes (13%) compared to controls (12%) (OR 2.3, 95% CI: 1.0–5.3). The hip in tennis players and runners was more prone to developing osteophytes (9.0%) compared to controls (4.0%) and had an almost 2.5-fold risk compared to controls (OR 2.5, 95% CI: 1.1–5.8). The development of joint space narrowing in the hip was also highest in tennis players and runners (12%) compared to controls (7.9%). Tennis players and runners had a 1.7-fold risk of joint space narrowing in the hip compared with controls (OR 1.7, 95% CI: 0.8–3.4). Most radiographic findings indicated early disease of OA comparable with grade 1 changes (mild, but definite disease) in both types of sport. Only two of the athletes had the most severe grade of osteophytes in the tibiofemoral joint (11%), four had grade 2 (22%), and twelve (67%) had grade 1 changes. The distribution of severity was similar at the other anatomical sites investigated. Tennis players showed a greater rate of osteophytes in the tibiofemoral joint (35%) than runners (19%). A similar rate of osteophytes in the hip was found between tennis players (14%) and runners (7.8%). In contrast, runners showed almost double the rate of joint space narrowing and osteophytes in the patellofemoral joint (45%) and in the hip (15%) compared with tennis players (29% and 7.1%, respectively) [51].

The study conducted by Lane et al. (1986) investigated the association between long-term and long-distance running and OA development [53]. A total of 41 runners (*n* = 18 females) were recruited through the 50-Plus Runners Association and were compared with a control group consisting of a community sample of 41 runners (*n* = 18 females). A questionnaire was used to collect data on multiple variables including medical history (estrogen replacement, age of menopause etc.), musculoskeletal injuries, and dietary history. Radiography of the lateral spine, knee, and hand (distal interphalangeal joint) was assessed. Measurements of osteophytes, sclerosis, and joint space narrowing were obtained and scored in increasing severity (0–3) using the KL scale (Table S3—Supplementary Materials). Furthermore, clinical signs of OA including Schober’s test, Heberden’s nodes, neck and knee crepitation, and knee instability were evaluated.

The study found no significant difference in the distal interphalangeal joint for sclerosis, osteophytes, and joint space narrowing between runners and the control group. Interestingly, runners were significantly more prone to developing both sclerosis and osteophytes in the knee compared to the control group. There was no significant difference reported for joint space narrowing in the knee between the two groups. The lumbar spine in runners was more prone to develop sclerosis compared to the control group. There was no significant difference reported for osteophytes and joint space narrowing at the lumbar spine between the two groups. Finally, there was no difference in clinical signs of OA between runners and the control group [53].

In summary, these findings suggest that former elite tennis and both former elite running and active non-elite running are associated with an increased radiographic presence of early signs of OA in weight bearing joints compared to controls.

### 3.5. Long-Distance Cross-Country Skiers

The study conducted by Michaëlsson et al. (2011) investigated the risk between long-distance cross-country skiing and knee and hip OA [54]. Both males (*n* = 48,574) and females (*n* = 5409) were recruited if they had finished the 90 km ski race Vasaloppet at least once in 1989 or between 1991 and 1998. Most of the included participants were not competing at an elite level. Data on finishing time, year of the race, and personal identification records were provided by the Vasaloppet office. Demographic data and diagnoses of hip and knee OA were obtained. The diagnosis of OA was defined by the presence of any surgical procedure involving arthroplasty and these data were extracted through matching their personal identification records with data from The Swedish National Patient registry. The number of person-years was determined individually for all participants in the study. It should be noted that the study did use pooled data (females and males combined) for some of the statistical analyses, hence it was not possible to extract results exclusively from females only for all parameters investigated.

The study found an incidence of 42 females (0.78%) who were treated with arthroplasty of the knee or hip after participating in Vasaloppet. Most participants (females and males) completed the ski race only once. Participants who had completed five or more races had a 70% higher rate of OA (hazard rate (HR): 1.7, 95% CI: 1.3–2.2) compared to participants who had only finished the race once. Moreover, a faster finishing time was also associated with an increased risk of OA (HR: 1.5, 95% CI: 1.1–2.0) compared to completing the race with a slow finishing time. Finally, finishing ≥5 races with a fast racing time was associated with an increased risk of OA (HR 2.7, 95% CI: 1.8–4.2) compared to the participants who completed the race once with a slow finishing time [54].

In summary, these findings suggest that non-elite cross-country skiers have a low incidence of arthroplasty, but participating in more races and having a faster finishing time is associated with a greater risk of being treated with arthroplasty for OA.

### 3.6. Risk of Bias Assessment

Risk of bias was evaluated and assessed from information such as sample size justification, whether exposure measures were consistent across all study participants and whether the recruited participants were selected from a similar population, and, finally, if potential confounders were considered in the study design and data analyses. The studies included were deemed to have an acceptable or fair risk of bias using the two assessment tools from NIH and SIGN (Table 3). A detailed assessment of the risk of bias score for each included study is available in Table S4—Supplementary Materials.

## 4. Discussion

The current systematic review evaluated the association between participation in popular sports in Europe or North and South America and the development of OA at different joints in females. The risk of developing OA for females participating in sports at both elite and non-elite levels and the impact of sex and sports participation is discussed below, along with suggestions for additional recommendations and future perspectives for this field.

### 4.1. Female Elite Sports as a Risk Factor for OA

It is a common concern that elite athletes are more prone to wear out their joints and develop OA [56]. However, little research has been dedicated to investigating the association between sports participation and the risk of OA in female athletes. The present systematic review found evidence to support that participating in elite sports is associated with the development of OA or the presence of early radiological and MRI signs compatible with OA in females. It should be noted that six of the included studies reported substantial sports-related injuries among the participants [46,47,48,49,51,52]. Therefore, it remains difficult to isolate the true effect of elite sports participation for the development of OA, since sports-related injuries are a well-known risk factor for the disease [57]. Only two of the included studies investigated female elite sports participants and OA compared with a control group [51,52]. These studies demonstrated an increased risk of OA development in weight bearing joints for those who had participated in long-term elite sports, including ballet, tennis, and running. Two other studies (Cooper et al., 2018 and 2021) investigated retired female athletes who had participated in the Olympic Games from UK and reported a prevalence of OA ranging from 26–35% [46,47]. The difference in the prevalence of OA between the two studies was most likely due to the latter study examining five joints (cervical and lumbar spine, hand, ankle, and foot) more than the first study by Cooper et al. from 2018 [46]. Nevertheless, these results show an increased risk of OA when compared to a large sample of medical records representing the general UK population of females [58]. In this population and at the age of 50 to 54 years, the average prevalence of OA was only reported to be approximately 7%, and for those aged 55 to 59 years the prevalence was approximately 13% in any joint among females.

OA was also commonly found in active elite female athletes competing in several sports. Boeth et al. (2017) reported that an average of 68% German female adolescent and adult elite volleyball players showed signs of early knee OA after two years of follow-up [49]. These athletes showed an increased risk of OA when compared to females of similar ages obtained from a large survey with randomly selected individuals from Germany [59]. The study reported an OA prevalence at any joint of 0.9% for early adulthood (18–29 years) and 4.3% for adults (30–44 years). Similar to the study by Boeth et al. (2017) [49], Angioi et al. (2014) found an average of 64% of UK female adult elite ballet dancers demonstrated early signs of OA in several weight bearing joints [50]. These athletes showed an increased risk of OA when compared to an approximately age-matched UK female reference population, where the prevalence of OA was only 0.2% at any joint [58]. Moreover, Merritt et al. 2021 reported that an average of 16% had OA among active female athletes competing in different sports during the Summer Olympics in 2016 [48]. This result shows an increased risk of OA when compared to the studies by Swain et al. (2020) and Fuchs et al. (2017) [58,59], which assessed the prevalence of OA in the general population in UK and Germany, respectively. It should be noted that elite sports participants could be represented in these two samples, but most likely only make up a small part of their total sample. Furthermore, variations in the prevalence of OA have been reported to differ between ethnicities and geographical disparities [60], but this would most likely not change that there is a substantial increase in OA among Olympic athletes compared to the background population.

Finally, the present systematic review found evidence that elite sports participation is associated with surgical treatment of OA. The two studies by Cooper et al. (2018 and 2021) found that 4.5–7.6% of retired Olympic athletes had undergone surgery for either hip or knee arthroplasty [46,47]. This result shows an increased risk of surgical treatment for OA compared to an estimated prevalence of 0.9% for hip arthroplasty and 1.5% for knee arthroplasty compared to a general female population in the US [61].

The increased risk of OA and arthroplasty in different sports could be explained by the fact that the elite athletes had competed for several years with high volume of training encountered throughout the years. The studies by Angioi et al. (2014) [50], Boeth et al. (2017) [49], and Niek Van Dijk et al. (1995) [52] reported that the athletes spend between 24–45 h per week training. Repetitive movements such as acceleration and deacceleration, jumping, or rapid weight transferring are often used during sports training, which may cause wear and tear of the joints and promote the increased risk of OA seen in elite athletes. Others have suggested that when the articular surface is slowly loaded, the synovial fluid begins to move to effectively distribute the load on the cartilage. In contrast, when the load is applied quickly, as in elite level sports, it does not allow enough time for distribution of the synovial fluid, which increases the force applied to the cartilage and this might cause joint damage [32,62].

### 4.2. Female Non-Elite Sports as a Risk Factor for OA

Previous studies have suggested prolonged endurance sports might lead to OA by overuse of joints [63]. The present systematic review found modest evidence to support that females who participate in non-elite endurance sports are more prone to the development of early radiological signs of OA. The study conducted by Lane et al. (1986) found an increased risk of early OA development in long-distance runners compared to a control group [53]. The female long-distance runners had covered 7796 km over the last 9.2 years, corresponding to approximately 6 km per day. Long-distance running requires a high-volume of training and is often practiced on hard surfaces; in addition, runners encounter several hills during training, which results in acceleration and deacceleration, thereby causing the knee joints to deteriorate and possibly initiate OA development. Interestingly, another systematic review found that long exposure (>15 years) with high-intensity and/or high-volume running was associated with an increased risk of knee OA [64]. However, it should be noted the authors could not determine whether the association was confounded by previous injuries [64]. Nevertheless, the exact threshold of training volume and intensity still needs to be elucidated further to fully understand its mechanism and how it influences the onset of OA.

Finally, the present systematic review found no clear evidence of a higher risk of surgery for OA in non-elite sports participation when compared to the general Swedish female population [65,66]. This could be explained by an age difference between participants in the Vasaloppet and those from the study of OA in the Swedish population who were above 45 years old. It could be speculated that most of the participants in the Vasaloppet study by Michaëlsson et al. (2011) were younger and less likely to be treated with surgery for OA [54].

### 4.3. The Impact of Sex and Sports Participation as a Risk Factor of OA

Females are generally at higher risk of developing OA compared to males, but the underlying mechanism is still not fully understood [67]. It has been suggested that anatomical differences between females and males might play an important role in the development of OA [17]. Females have smaller joint surfaces and decreased cartilage volume, and lose cartilage at a higher annual rate compared to males [68,69]. Little research efforts have been dedicated to investigating the association between sports participation and the risk of OA between sexes. Surprisingly, the present systematic review found no clear difference between females and males participating in sports and the risk of developing OA [48,49,54]. However, male elite athletes tended to have a higher prevalence of OA than female elite athletes in the study conducted by Merritt et al. (2021) [48]. Although speculative, this tendency could be explained by the gap in sports performance since male athletes are superior at running, jumping, and throwing compared to female athletes resulting in greater load applied to their joints [70]. Moreover, Cooper et al. (2018) reported that male elite athletes had a higher body mass index just below the threshold for being overweight compared to female elite athletes [46]. Being overweight has been suggested to be associated with a 3-fold increased risk of developing OA.

Similar to the study by Michaëlsson et al. (2011) [54], the trend of a higher incidence of OA in non-elite skiing males compared with females might, to some degree, be explained by differences in their body weight. However, it should be noted that the study did not report these data, and thus the explanation is rather speculative.

### 4.4. Strengths and Limitations

The strengths of the present systematic review were warranted by an appropriate methodology using a systematic search strategy to harness data from two major academic publishing databases (PubMed and Embase). Additionally, Google Scholar was used as a supplementary database along with backwards and forwards citation chaining to find other relevant articles that did not exist in the two primary databases. Three authors were contacted by mail to provide specific data on OA for females; unfortunately, no responses were received. Another strength of the review was that the included articles were thoroughly assessed for potential risk and bias and their overall research quality was assessed using the NIH and SIGN tools.

The present systematic review also has some limitations. Even though the risk of bias was considered fair and acceptable, four of the included studies used relatively small sample sizes [49,50,52,53], which can undermine the external validity of their findings and the present systematic review [71]. Only a few of the studies included had explicitly investigated female sports participation and its association with OA, and some of the articles had only a rather small segment dedicated to the topic. Furthermore, only two of the included studies used a follow-up period, thus making it difficult to assess the protentional onset or temporal progression of the disease. Consequently, other study designs than prospective or longitudinal were included to answer the main aim of the present systematic review and to provide greater depth of insight.

It should be noted that although the risk of bias assessment was conducted thoroughly using already established and validated bias assessment tools (NHI and SIGN), it is an inherent limitation to the present systematic review that only one investigator conducted the assessment. Similarly, it is a limitation that the screening of identified articles and the subsequent extraction process was conducted by a single reviewer (MB). However, in order to retain a high sensitivity, a prioritized list of exclusion criteria was used and a standardized data extraction template [72]. Another important limitation is the large heterogeneity in age among the included female athletes and sports participants in the included studies of the present review. The age across all sports disciplines ranged from 15 to above 60 years. However, we decided not to impose age restrictions when assessing studies for inclusion in order to ensure a broad age-related representation. Unfortunately, not all of the included studies provided information about body composition or BMI (only five out of nine studies provided data on BMI). Since body composition and BMI are well-known risk factors for the development of osteoarthritis, it cannot be ruled out that at least part of the increased risk of developing OA from sports participation is directly linked to body composition or BMI.

Finally, most of the included studies did not use a proper control group, hence data from patient registries and studies on OA among the general population was used for comparison whenever appropriate.

### 4.5. Future Recommendations and Perspectives

Based on the findings from the present systematic review, physicians and sports coaches may consider informing both active and retired elite sports athletes and non-elite sports participants about the increased risk of developing OA in sports and provide education about the disease and information about different treatment strategies. However, this should not be viewed as a warning for not continuing an active lifestyle since sports participation promotes several other health benefits [73].

It should be noted that large studies investigating female sports participation and OA were lacking, especially in the field of female non-elite sports. Moreover, there was an absence of studies investigating several different types of popular sports such as handball, wrestling, and football. Studies in males have suggested that these types of sports are associated with a higher risk of OA [31,32]. This highlights the need for future research efforts to examine whether females participating in handball, wrestling, and football have an increased risk of developing OA as well.

## 5. Conclusions

The present systematic review found that female elite sports participation was associated with a higher risk of OA compared to either controls or the general population. Additionally, female elite sports participation was associated with a higher risk of receiving surgical treatment for OA when compared to the general population. The review also found that female non-elite sports participation was associated with a higher risk of OA development when compared to controls. However, female non-elite sports participation did not have an increased risk of surgical treatment for OA when compared to the general population.

Finally, the systematic review also found no clear differences between the impact of sex and elite sports participation and OA development or non-elite sports participation and end-stage OA. Isolating the true effect of sports participation on the development of OA remains difficult as injuries are common in sports participation. Sports coaches and physicians are recommended to inform about the increased risk of OA to athletes and sports participants.

## Figures and Tables

**Table 1:** Demographics in the included studies. [Square brackets] represent combined male and female data. {Curly brackets} represent ranges for females alone. Not reported (NR). Data are represented as mean ± SD (if not otherwise specified).

First Author, Year	Sample Size, Females (*n*)	Age, (years)	BMI (kg/m^2^)	Duration of Competitive Career, (Years)	Age When Starting to Compete, (Years)	Age When Ceasing to Compete, (Years)	Hip or Knee Arthroplasty, (%)
Merritt, 2021 [48]	160	34% <25 41% 25–29 24% ≥30	NR	NR	NR	NR	NR
Cooper, 2021 [47]	274	54 {23–93}	23.7 ± 4.7	10.2 ± 6.8	NR	NR	4.5
Cooper, 2018 [46]	244	59.0 ± 12.2	23.8 ± 4.0	9.2 ± 5.4	18.5 ± 4.8	27.5 ± 7.3	7.6
Boeth, 2017 [49]	19	Adolescents: [16.0 ± 0.8] Adults: [46.8 ± 5.1]	Adolescents: [21.50 ± 1.79] Adults: [24.41 ± 2.91]	>3 years of participation in a club	NR	NR	NR
Angioi, 2014 [50]	11	28.3 ± 5.0	NR	>5 years of professional ballet	NR	NR	NR
Michaëlsson, 2011 [54]	5,409	15 to 60+	NR	NR	NR	NR	0.78
Spector, 1996 [51]	81	52.3 ± 6.1	22.1 ± 2.8	Runners: 15.3 Tennis: 19.3	NR	NR	NR
Niek Van Dijk, 1995 [52]	19	59 {50–66}	NR	37 {13 to 54}	NR	NR	NR
Lane, 1986 [53]	18	57.7	NR	[9.2]	NR	NR	NR

**Table 2:** Characteristics and main findings of the included studies. Abbreviations used: Osteoarthritis (OA), whole-organ magnetic resonance imaging score (WORMS), Kellgren–Lawrence scale (KL), magnetic resonance imaging (MRI), and not specified (NS).

First Author, Year	Diagnosis of OA	Anatomical Joints Assessed	Type of Sport, Performance Level	Main Findings
Merritt, 2021 [48]	MRI (Delphi exercise)	Knee, hip, shoulder, elbow, ankle, and wrist	Sport not specified, elite	16% had OA at any joint. The severity assessed included: 3.8% mild, 4.4% moderate, and 7.5% had severe OA.
Cooper, 2021 [47]	Questionnaire	Cervical and lumbar spine, hand, hip, knee, ankle, and foot	Sport not specified, elite	35% had OA at any joint. This included: 1.9% in cervical spine, 7.5% in lumbar spines, 2.2% in 1st carpometacarpal joint, 7.8% in hips, 12% in knees, 7.8% in hips, 2.2% in ankles, and 1.9% in 1st metatarsophalangeal joint.
Cooper, 2018 [46]	Questionnaire	Knee and hip	Sport not specified, elite	26% had OA at any joint.
Boeth, 2017 [49]	MRI (WORMS)	Knee	Volley ball athletes, elite	68% showed early signs of knee OA. No differences in the prevalence and severity of knee abnormalities between adolescent and adult volleyball players after 2 years of follow-up.
Angioi, 2014 [50]	MRI (KL)	Knee, hip, ankle, foot, spine	Ballet, elite	64% showed early signs of OA in different joints. The distribution of OA was 71% in knees, 14.3% in the spine, and 14% in hip.
Michaëlsson, 2011 [54]	National Patient Registry for identification of diagnoses	Knee and hip	Cross-country skiing, non-elite	A total of 42 (0.8%) developed OA (joint not specified) over a period of 9 years.
Spector, 1996 [51]	data Radiography (Scale similar to KL)	Knee and hip	Middle- and long-distance runners and tennis players, ex-elite	Participation in weight bearing sports was associated with increased risk (2–2.7-fold) of radiologic OA.
Niek Van Dijk, 1995 [52]	Radiography (Modified scale of Hermodsson)	Hip, ankle, and foot	Ballet, elite	All joints assessed demonstrated a tendency of increased risk of OA in ballet dancers compared to controls. A total of 38 hips and first metatarsophalangeal joints showed the highest severity of OA respectively 5.3% and 12%.
Lane, 1986 [53]	Radiography (NS)	Knee, hand, and spine	Long distance runners, non-elite	Radiographic signs of early OA including sclerosis and spurs were more present in long distance runners compared to a control group.

**Table 3:** Results of the risk of bias assessment for the included studies. National Health Institute (NHI). Scottish Intercollegiate Guidelines Network (SIGN).

First Author, Year	Study Design	Assessment Tool	Score and Evaluation
Merritt, 2021 [48]	Cross-sectional	NHI	7 (fair risk of bias)
Cooper, 2021 [47]	Cross-sectional	NHI	9 (fair risk of bias)
Cooper, 2018 [46]	Cross-sectional	NHI	9 (fair risk of bias)
Boeth, 2017 [49]	Longitudinal cross-sectional	NHI	9 (fair risk of bias)
Angioi, 2014 [50]	Cohort	NHI	8 (fair risk of bias)
Michaëlsson, 2011 [54]	Cohort	NHI	8 (fair risk of bias)
Spector, 1996 [51]	Case-control	SIGN	10 (acceptable)
Niek Van Dijk, 1995 [52]	Case-control	SIGN	8 (acceptable)
Lane, 1986 [53]	Case-control	SIGN	6 (acceptable)

## Data Availability

The authors confirm that the data supporting the findings of this study are available within the article and its supplementary materials.

## Supplementary Materials

The following supporting information can be downloaded at: https://www.mdpi.com/article/10.3390/sports12010015/s1; Table S1: The template of the National Institutes of Health (NIH) quality assessment tool (https://www.nhlbi.nih.gov/health-topics/study-quality-assessment-tools, accessed 1 December 2022); Table S2: The template of the Scottish Intercollegiate Guidelines Network (SIGN) quality assessment tool (https://www.sign.ac.uk/what-we-do/methodology/checklists/, accessed 1 December 2022); Table S3: The Kellgren-Lawrence scale. Adapted from: Kellgren JH. Radiological signs of rheumatoid arthritis; a study of observer differences in the reading of hand films; Table S4: Results of the risk of bias assessment for the included studies. National Health Institute (NHI). Scottish Intercollegiate Guidelines Network (SIGN). Ref. [74] is cited in supplementary materials.

## Appendix A

**Table A1:** Schematic overview of search string including blocks and keywords used to systematically search PubMed and Embase. No formal search string was developed for Google Scholar, but the same keywords presented in Block 1 and Block 2 were used in various combinations. * denotes truncation symbol.

	PubMed	Embase
Block 1	woman[Mesh] OR women[Mesh] OR female[Mesh] OR girl[Mesh]	exp woman/OR exp women/OR exp female/OR exp girl/
Block 2	osteoarthritis[Mesh] OR arthrosis[tw] OR osteoarthrit*[tw] OR osteo-arthrit*[tw] OR osteoarthro*[tw] OR osteo-arthro*[tw] OR arthrosis[tw] OR arthroses[tw] OR arthrot*[tw]	exp osteoarthritis/OR arthrosis.tw. OR osteoarthrit*.tw. OR osteo-arthrit*.tw. OR osteoarthro*.tw. OR osteo-arthro*.tw. OR arthrosis.tw. OR arthroses.tw. OR arthrot*.tw.
Block 3	“american football”[tw] OR football[tw] OR NFL[tw] OR handball[tw] OR “ice hockey”[tw] OR wrestling[tw] OR shooting[tw] OR bobsleigh[tw] OR baseball[tw] OR archery[tw] OR curling[tw] OR “speed skating”[tw] OR skeleton[tw] OR Soccer[tw] OR Golf[tw] OR running[tw] OR runner[tw] OR “cross country”[tw] OR marathon[tw] OR sprint[tw] OR steeplechase[tw] OR Athletics* OR “track and field” OR javelin[tw] OR “hammer throw”[tw] OR hurdles[tw] OR “long jump”[tw] OR “high jump”[tw] OR “pole vault”[tw] OR bowls[tw] OR tennis[tw] OR badminton[tw] OR squash[tw] OR Racquetball[tw] OR rugby[tw] OR cricket[tw] OR Equestrian OR “horse-riding” OR dressage OR “show jumping” OR hockey[tw] OR netball[tw] OR basket[tw] OR swimming[tw] OR diving[tw] OR “water polo”[tw] OR sailing[tw] OR angling[tw] OR cycling[tw] OR boxing[tw] OR weightlifting[tw] OR “snow sports” OR skiing[tw] OR snowboarding[tw] OR skating[tw] OR ”table tennis”[tw] OR mountaineering[tw] OR rowing[tw] OR gymnastics[tw] OR volleyball[tw] OR taekwondo[tw] OR dancing[tw] OR rounders[tw] OR judo[tw] OR fencing[tw] OR sport[tw]	‘american football’.tw. OR football.tw. OR NFL.tw. OR handball.tw. OR ‘ice hockey’.tw. OR wrestling.tw. OR shooting.tw. OR bobsleigh.tw. OR baseball.tw. OR archery.tw. OR curling.tw. OR ‘speed skating’.tw. OR skeleton.tw. OR soccer.tw. OR golf.tw. OR run*.tw. OR ‘cross country ‘.tw. OR marathon.tw. OR sprint.tw. OR steeplechase.tw. OR athletics* OR ‘track and field’ OR javelin.tw. OR ‘hammer throw’.tw. OR hurdles.tw. OR ‘long jump’.tw. OR ‘high jump’.tw. OR ‘pole vault’.tw. OR bowls.tw. OR tennis.tw. OR badminton.tw. OR squash.tw. OR racquetball.tw. OR rugby.tw. OR cricket.tw. OR equestrian OR ‘horse-riding’ OR dressage OR ‘show jumping’ OR hockey.tw. OR netball.tw. OR basket.tw. OR swimming.tw. OR diving.tw. OR ‘water polo’.tw. OR sailing.tw. OR angling.tw. OR cycling.tw. OR boxing.tw. OR weightlifting.tw. OR ‘snow sports’ OR skiing.tw. OR snowboarding.tw. OR skating.tw. OR ‘table tennis’.tw. OR mountaineering.tw. OR rowing.tw. OR gymnastics.tw. OR volleyball.tw. OR taekwondo.tw. OR dancing.tw. OR rounders.tw. OR judo.tw. OR fencing.tw. OR exp sports/
Block 4	Spine[tw] OR elbow[tw] OR shoulder[tw] OR neck[tw] OR hand[tw] OR ankle[tw] OR knee[tw] OR hip[tw]	spine.tw. OR elbow.tw. OR shoulder.tw. OR neck.tw. OR hand.tw. OR ankle.tw. OR knee.tw. OR hip.tw.
Block 5	Prospective[tw] OR longitudinal [tw]	prospective.tw. OR longitudinal .tw.
