# Supplementary material for: Sports Participation and Osteoarthritis in Females: A Systematic Review
Source: Sports (Basel). 2023 Dec 31;12(1):15. doi: 10.3390/sports12010015 (PMC10819498; doi:10.3390/sports12010015)
Supplement: Supplementary file 1 [file sports-12-00015-s001.zip › Supplementary_material_revision_2.pdf]

Sports participation and osteoarthritis in females: A systematic review

Supplementary material

Table of Contents:

- Table S1. The template of The National Institutes of Health (NIH) quality assessment tool (https://www.nhlbi.nih.gov/health-topics/study-quality-assessment-tools).
- Table S2. The template of the Scottish Intercollegiate Guidelines Network (SIGN) quality assessment tool (https://www.sign.ac.uk/what-we-do/methodology/checklists/).
- Table S3. The Kellgren-Lawrence scale. Adapted from: Kellgren JH. Radiological signs of rheumatoid arthritis; a study of observer differences in the reading of hand films. *Ann Rheum Dis.* 1956 Mar;15(1):55–60.
- Table S4. Results of the risk of bias assessment for the included studies. National Health Institute (NHI). Scottish Intercollegiate Guidelines Network (SIGN).

Table S1. The template of The National Institutes of Health (NIH) quality assessment tool (https://www.nhlbi.nih.gov/health-topics/study-quality-assessment-tools, accessed 1 December 2022).

Was the research question or objective in this paper clearly stated
Was the study population clearly specified and defined
Was the participation rate of eligible persons at least 50%
Were all the subjects selected or recruited from the same or similar populations (including the same time period)? Were inclusion and exclusion criteria for being in the study prespecified and applied uniformly to all participants
Was a sample size justification, power description, or variance and effect estimates provided
For the analyses in this paper, were the exposure(s) of interest measured prior to the outcome(s) being measured
Was the timeframe sufficient so that one could reasonably expect to see an association between exposure and outcome if it existed
For exposures that can vary in amount or level, did the study examine different levels of the exposure as related to the outcome (e.g., categories of exposure, or exposure measured as continuous variable)
Were the exposure measures (independent variables) clearly defined, valid, reliable, and implemented consistently across all study participants
Was the exposure(s) assessed more than once over time<br>Were the outcome measures (dependent variables) clearly defined, valid, reliable, and implemented consistently across all study participants
Were the outcome assessors blinded to the exposure status of participants
Was loss to follow-up after baseline 20% or less
Were key potential confounding variables measured and adjusted statistically for their impact on the relationship between exposure(s) and outcome(s)

Table S2. The template of the Scottish Intercollegiate Guidelines Network (SIGN) quality assessment tool (https://www.sign.ac.uk/what-we-do/methodology/checklists/, accessed 1 December 2022).

The study addresses an appropriate and clearly focused question
The cases and controls are taken from comparable populations
**SELECTION OF SUBJECTS**
The same exclusion criteria are used for both cases and controls
What percentage of each group (cases and controls) participated in the study
Comparison is made between participants and non-participants to establish their similarities or differences
Cases are clearly defined and differentiated from controls
It is clearly established that controls are non-cases
**ASSESSMENT**
Measures will have been taken to prevent knowledge of primary exposure influencing case ascertainment
Exposure status is measured in a standard, valid and reliable way
**CONFOUNDING**
The main potential confounders are identified and taken into account in the design and analysis
**STATISTICAL ANALYSIS**
Confidence intervals are provided
**OVERALL ASSESMENT OF THE STUDY**
How well was the study done to minimise the risk of bias or confounding
Taking into account clinical considerations, your evaluation of the methodology used, and the statistical power of the study, do you think there is clear evidence of an association between exposure and outcome
Are the results of this study directly applicable to the patient group targeted by this guideline

Table S3. The Kellgren-Lawrence scale. Adapted from: Kellgren JH. Radiological signs of rheumatoid arthritis; a study of observer differences in the reading of hand films [1].

Grade	Description
0	No presence of OA
1	Possible osteophytic lipping, doubtful joint space narrowing
2	Possible joint space narrowing, definite osteophytes
3	Possible deformity of bone ends, some sclerosis, moderate multiple osteophytes, definite joint space narrowing
4	Definite bony end deformity, large osteophytes, severe sclerosis, marked joint space narrowing

Table S4. Results of the risk of bias assessment for the included studies. National Health Institute (NHI). Scottish Intercollegiate Guidelines Network (SIGN).

SIGN	1	2	3	4	5	6	7	8	9	10	11	12	13	14	15	Total
Spector, 1996 [2]	Yes	Yes	No	No	Yes	Yes	Yes	Yes	No	Yes	Yes	Yes	No	No	Yes	10
Niek Van Dijk, 1995 [3]	Yes	Yes	Yes	No	Yes	Yes	Yes	No	Yes	No	Yes	No	No	No	No	8
Lane, 1986 [4]	Yes	Yes	No	Yes	Yes	Yes	Yes	No	No	No	No	No	No	No	No	6
NHI	1	2	3	4	5	6	7	8	9	10	11	12	13	14	Total	
Merritt, 2021 [5]	Yes	Yes	Yes	Yes	No	No	Yes	No	No	No	Yes	Yes	No	No	7	
Cooper, 2021 [6]	Yes	Yes	Yes	No	Yes	Yes	Yes	No	No	No	Yes	No	No	Yes	9	
Cooper, 2018 [7]	Yes	Yes	Yes	Yes	Yes	Yes	Yes	No	No	No	Yes	No	No	Yes	9	
Boeth, 2017 [8]	Yes	Yes	Yes	No	No	Yes	Yes	No	Yes	Yes	Yes	No	Yes	No	9	
Angioi, 2014 [9]	Yes	Yes	Yes	Yes	No	Yes	Yes	No	Yes	No	Yes	No	No	No	8	
Michaëls-son, 2011 [10]	Yes	Yes	Yes	No	No	Yes	Yes	No	Yes	Yes	Yes	No	No	No	8	

References

1. Kellgren, J.H.; Bier, F. Radiological Signs of Rheumatoid Arthritis: A Study of Observer Differences in the Reading of Hand Films. *Ann Rheum Dis* **1956**, *15*, 55, doi:10.1136/ARD.15.1.55.

2. Spector, T.D.; Harris, P.A.; Hart, D.J.; Cicuttini, F.M.; Nandra, D.; Etherington, J.; Wolman, R.L.; Doyle, D. V. Risk of Osteoarthritis Associated with Long-Term Weight-Bearing Sports: A Radiologic Survey of the Hips and Knees in Female Ex-Athletes and Population Controls. *Arthritis Rheum* **1996**, *39*, 988–995, doi:10.1002/ART.1780390616.

3. Niek Van Dijk, C.; Lim, L.S.L.; Poortman, A.; Strübbe, E.H.; Marti, R.K. Degenerative Joint Disease in Female Ballet Dancers. *Am J Sports Med* **1995**, *23*, 295–300, doi:10.1177/036354659502300307.

4. Lane, N.E.; Bloch, D.A.; Jones, H.H.; Marshall, W.H.; Wood, P.D.; Fries, J.F. Long-Distance Running, Bone Density, and Osteoarthritis. *JAMA* **1986**, *255*, 1147–1151, doi:10.1001/JAMA.1986.03370090069022.

5. Merritt, A.; Roemer, F.W.; Heiss, R.; Jarraya, M.; Guermazi, D.; Hayashi, D.; Engebretsen, L.; Crema, M.D.; Guermazi, A. Frequency of MRI-Detected Peripheral Osteoarthritis in Athletes during the Summer Olympics in Rio 2016. *Osteoarthr Cartil Open* **2021**, *3*, doi:10.1016/J.OCARTO.2021.100199.

6. Cooper, D.J.; Batt, M.E.; O'Hanlon, M.S.; Palmer, D. A Cross-Sectional Study of Retired Great British Olympians (Berlin 1936-Sochi 2014): Olympic Career Injuries, Joint Health in Later Life, and Reasons for Retirement from Olympic Sport. *Sports Med Open* **2021**, *7*, doi:10.1186/S40798-021-00339-1.

7. Cooper, D.J.; Scammell, B.E.; Batt, M.E.; Palmer, D. Factors Associated with Pain and Osteoarthritis at the Hip and Knee in Great Britain’s Olympians: A Cross-Sectional Study. *Br J Sports Med* **2018**, *52*, 1101–1108, doi:10.1136/BJSPORTS-2017-098315.

8. Boeth, H.; MacMahon, A.; Eckstein, F.; Diederichs, G.; Schlausch, A.; Wirth, W.; Duda, G.N. MRI Findings of Knee Abnormalities in Adolescent and Adult Volleyball Players. *J Exp Orthop* **2017**, *4*, doi:10.1186/S40634-017-0080-X.

9. Angioi, M.; Maffulli, G.D.; McCormack, M.; Morrissey, D.; Chan, O.; Maffulli, N. Early Signs of Osteoarthritis in Professional Ballet Dancers: A Preliminary Study. *Clin J Sport Med* **2014**, *24*, 435–437, doi:10.1097/JSM.0000000000000035.

10. Michaëlsson, K.; Byberg, L.; Ahlbom, A.; Melhus, H.; Farahmand, B.Y. Risk of Severe Knee and Hip Osteoarthritis in Relation to Level of Physical Exercise: A Prospective Cohort Study of Long-Distance Skiers in Sweden. *PLoS One* **2011**, *6*, doi:10.1371/JOURNAL.PONE.0018339.
